# A Comparative Study of Choline Chloride Deep Eutectic Electrolytes: Towards Sustainable Supercapacitors

**DOI:** 10.3390/molecules31060929

**Published:** 2026-03-11

**Authors:** Raquel San Emeterio, Antía Santiago-Alonso, Juan José Parajó, Ana T. S. C. Brandão, Carlos M. Pereira, Carlos Gracia, Pablo Vallet, Renata Costa, Josefa Salgado

**Affiliations:** 1 NaFoMat, Department of Applied Physics/Materials Institute of USC (iMATUS), Universidade de Santiago de Compostela, Campus Vida, 15782 Santiago de Compostela, Spain; raquel.sanemeterio@usc.es (R.S.E.); antia.santiago.alonso@usc.es (A.S.-A.); 2 Centro de Investigación en Tecnologías Navales e Industriales, Universidade de A Coruña, Campus Industrial de Ferrol, 15403 Ferrol, Spain; juan.jose.parajo.vieito@udc.es; 3 Chemistry Research Centre of the University of Porto/Institute of Molecular Sciences (CIQUP-IMS), Faculty of Sciences, University of Porto, Rua do Campo Alegre s/n, 4169-007 Porto, Portugal; ana.brandao@fc.up.pt (A.T.S.C.B.);; 4 TA Instruments Waters Chromatography, Tres Cantos, 28760 Madrid, Spain; carlos_gracia@waters.com; 5 AVL Ibérica, Paseo Arco de Ladrillo 68, Floor n. 5, 47007 Valladolid, Spain

**Keywords:** DES, choline chloride, DSC, EIS, cyclic voltammetry, NMR, supercapacitor, glyceline, ethaline, reline

## Abstract

Over the past few decades, ionic liquids (ILs) have gained attention as electrolytes, although concerns about their environmental persistence and toxicity challenge their status as green solvents. In this framework, choline chloride (ChCl) offers a more sustainable alternative due to its low toxicity, biodegradability, and cost-effectiveness. Although ChCl has a high melting point, its combination with hydrogen bond donor compounds (HBDs) can result in liquid mixtures at much lower temperatures, known as deep eutectic solvents (DESs). This study presents a comparative evaluation of three ChCl-based DESs, glyceline, ethaline, and reline (obtained from mixtures of ChCl and glycerol, ethylene glycol, and urea), with a focus specifically on their potential as electrolyte candidates for supercapacitors. Using differential scanning calorimetry (DSC), cyclic voltammetry (CV), electrochemical impedance spectroscopy (EIS), and nuclear magnetic resonance (NMR), we assess their thermal, electrochemical, and structural properties. All DESs displayed amorphous behavior and a strong tendency to remain liquid even at very low temperatures. Among them, ethaline showed the most promising electrochemical performance, exhibiting the lowest resistivity and the highest capacity.

## 1. Introduction

Ionic liquids (ILs) have been extensively studied as electrolytes due to their high ionic conductivity, wide electrochemical window, and molecular design flexibility. Owing to their negligible vapor pressure and non-flammability, ILs have traditionally been regarded as green solvents. Nevertheless, some studies have raised concerns about their true sustainability, since many of them are toxic, environmentally persistent, and require complex and energy-intensive synthesis processes compared to conventional solvents [1,2]. Therefore, to minimize the environmental impact of emerging energy storage technologies, the development and implementation of new eco-friendly and sustainable materials must be prioritized.

In the search for more environmentally friendly electrolytes, choline chloride (ChCl)-based systems have been proposed as a promising alternative. ChCl is a very common additive, used in animal and human nutrition, in the pharmaceutical industry and even in fertilizer production. It is characterized by low toxicity and high biodegradability, characteristics widely reported in the literature [3,4]. Moreover, ChCl-based compounds generally feature simpler and less energy-demanding synthesis routes, producing less waste, as Jesus and Marciel highlight in their recent review [2]. Although DESs are often presented as green solvent alternatives, recent studies have shown that their sustainability cannot be assumed a priori and must be evaluated on a case-by-case basis. For example, life-cycle assessment of reline (ChCl/urea) demonstrated that its environmental impacts depend largely on the upstream production of its constituent chemicals, while recent reviews have stressed the need for system-specific life-cycle and techno-economic analyses before assigning broad green credentials to DESs [5,6].

Despite these advantageous characteristics, ChCl has a melting point far above room temperature, which considerably limits its direct application [7]. To overcome this limitation, ChCl is usually combined with other compounds to form deep eutectic solvents (DESs). DESs consist of the combination of a Hydrogen Bond Acceptor (HBA)—in this case ChCl—and a hydrogen bond donor (HBD) in a specific ratio so that the mixture has a melting point significantly lower than its individual components.

DESs have numerous applications, including catalysis and reaction media, biomass separation and extraction processes, uses in the pharmaceutical and food industries, CO_2_ capture, and fuel desulphurization, among others [5]. Regarding energy storage, DESs have become, during the last few years, a leading class of beyond-aqueous electrolytes, mainly due to the combination of their low volatility and non-flammability with electrochemical stability windows that can exceed those of conventional aqueous systems. Specifically, mixtures of ChCl and different HBD solvents are commonly proposed for electrochemical applications. Examples of these HBD solvents include urea [6], ethylene glycol [7], malonic acid [8], and lactic acid [9], among others. From an environmental perspective, the selection of glycerol, ethylene glycol, and urea as HBDs—resulting in the DESs glyceline, ethaline, and reline—is particularly significant, as can be seen in the paper by Nejrotti et al. [8], who presented a sustainability assessment of six different HBDs in combination with ChCl.

Moving onto DES application in supercapacitors, the consensus is that performance is frequently transport-limited, with viscosity–conductivity trade-offs and micro-heterogeneity governing equivalent series resistance and rate capability, particularly in hydrogen-bonded liquids [9]. Additionally, these compounds exhibit high thermal stability, with degradation temperatures around 200 °C, ensuring reliable operation over a broad temperature range [10]. More broadly, systematic comparisons across Hydrogen Bond Acceptor/hydrogen bond donor identities and ratios confirm that subtle changes in hydrogen-bond topology can drive large shifts in melting/crystallization tendencies and ion mobility, directly impacting usable temperature windows and power performance [11,12].

At the electrode–electrolyte boundary, state-of-the-art understanding emphasizes that capacitance is influenced not only by bulk transport but also by potential-dependent ion packing, specific adsorption, and hydrogen-bond-mediated organization within the electrical double layer [13]. Recent studies involving differential capacitance assessment in DESs have highlighted both the scarcity of reliable datasets and the sensitivity of extracted capacitance to protocol choices, strengthening the case for complementary CV/EIS/differential capacitance reporting in comparative studies [14]. In parallel, molecular dynamics studies of ethaline at gold surfaces now provide mechanistic interpretations of how HBD identity affects layering and orientational ordering, offering a route to rationalize compact versus “structured” double layers observed experimentally [15]. Consistently, device-level demonstrations using reline have achieved stable high-potential carbon supercapacitors (∼2.2 V) with long cycle life, illustrating that DESs can enable higher operating potentials without resorting to flammable organic electrolytes [16].

Glyceline, ethaline and reline have been previously studied as electrolytes for supercapacitors [9,16,17,18,19,20], showing the following key findings: (i) ethaline stands out due to its relatively high ionic conductivity and low viscosity, (ii) glyceline is a good choice in terms of sustainability at the expense of a higher viscosity and (iii) reline is attractive due to its low cost although it generally exhibits poorer transport properties. However, most of the studies focus primarily on density, viscosity and electrical conductivity measurements, rather than their contribution to capacitance, which is one of the aspects addressed in this work. Agieienko et al. [18] point out that over 30 publications of these properties can be found for glyceline. These reported results vary significantly, highlighting the importance of comparative studies such as the present work, in which we evaluate the three DESs on the same testing platform, using identical electrode materials, cell architecture and measurement protocols. Overall, the current state of the art is converging on integrated, multiscale electrolyte design combining sustainability screening, rigorous thermophysical characterization, and molecular-level interfacial models to predict capacitance and resistance. Following this aim to link DES structure and properties, here we present a comparative study of three DESs (glyceline, ethaline and reline) which includes a thermal characterization through differential scanning calorimetry (DSC), an electrochemical performance study consisting of cyclic voltammetry (CV) and electrochemical impedance spectroscopy (EIS), and a structural analysis with nuclear magnetic resonance (NMR). Our main objective is to identify the most promising candidates and provide a clearer understanding of how the choice of HBD influences electrolyte performance. In addition, although the inclusion of a broader set of HBDs and different HBA ratios would further expand the compositional landscape of ChCl-based DES electrolytes, such an analysis lies beyond the scope of the present work. Here, we intentionally focus on three benchmark systems, glyceline, ethaline, and reline, in order to compare representative polyol- and amide-based ChCl DESs under identical experimental conditions and to isolate the effect of HBD identity. This focused scope is appropriate for the aims of the present study, namely to identify the most promising candidate for supercapacitor applications and to establish clear structure–property–performance trends within a controlled set of widely studied DESs. A broader compositional assessment, including additional polyols, dicarboxylic acids, and different ChCl molar ratios, has recently been investigated in complementary work by Costa et al. [12], where both HBD chemistry and composition were shown to substantially affect the interfacial electrochemical response.

## 2. Results

### 2.1. Differential Scanning Calorimetry (DSC)

**Figure 1:**
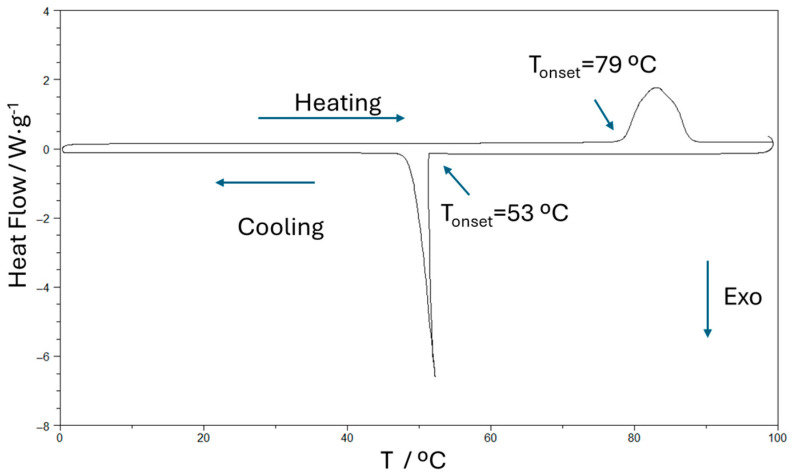
DSC curves of pristine choline chloride.

Figure 1 shows the DSC heating and cooling curves of pure choline chloride (ChCl) at 5 °C/min after two purification steps: maintaining the sample under vacuum for at least 24 h and performing an isothermal step at 125 °C for 45 min. The literature reports that ChCl has a high melting temperature, approximately 302–305 °C, but the substance is difficult to detect because it undergoes thermal decomposition upon melting. This thermal behavior reflects the strong ionic interactions present in its crystalline structure and limits its direct use [9]. The endothermic peak observed upon heating has an onset at 79 °C and an associated heat of 116 J·g^−1^ (or 16.2 kJ·mol^−1^). A corresponding exothermic peak appears during the cooling ramp at 53 °C, with an associated heat of 113 J·g^−1^. These signals are attributed to a solid–solid transition, as explained in the work of Petrouleas and Lemmon [21] in 1978 and subsequently analyzed in depth by Lobo et al. [7]. This supercooling effect of 25 °C is very usual in ILs and is associated with slow nucleation kinetics, strong ionic disorder, and Coulomb and directional interactions that hinder crystal reorganizations. Due to this behavior, a reflection of a flexible and disordered structure, this IL is a good candidate for DES formation, as small changes in the molecular environment can move melting to lower temperatures or even induce completely amorphous behavior.

**Figure 2:**
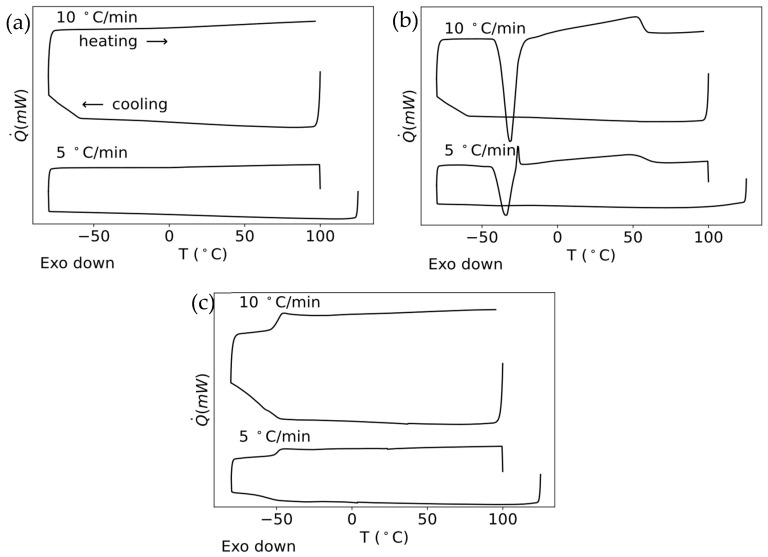
DSC scans at 10 °C·min^−1^ and 5 ° C·min^−1^ of (**a**) glyceline, (**b**) ethaline and (**c**) reline (offset vertically for clarity).

Figure 2 summarizes the DSC curves of heating and cooling at two different heating rates (5 and 10 °C/min) of the mixtures of choline chloride with the three selected HBDs in the proportion 1:2 (IL:HBD). These systems are typically referred to in the literature as glyceline (ChCl + glycerol), ethaline (ChCl + ethylene glycol) and reline (ChCl + urea) (see Materials and Methods Section for more details and information). It is important to highlight the significance of performing the DSC scans at two heating rates, as this approach allows for a more reliable interpretation and identification of thermal events.

In contrast to pure ChCl, the mixtures show radically different thermal behavior upon cooling, characterized by the total frustration of crystallization. This indicates a complete suppression of the ability of the system to organize into crystalline phases, suggesting the formation of a highly disordered amorphous liquid state stabilized by strong interactions between ionic liquid and solvent. Furthermore, glyceline and reline do not show endothermic or exothermic peaks upon heating; only a clear glass transition appears in reline at −49 °C (for both scanning rates used in this work). Glyceline probably also presents a glass transition at temperatures lower than those used in this work. This indicates that these mixtures (glyceline and reline) are highly disordered amorphous liquids, stabilized by strong interactions between the IL and the cosolvent.

In contrast, ethaline shows a single well-defined exothermic event at low temperatures and for both scanning rates (around −40 °C). This event corresponds to cold crystallization, because the mixture, which is in a metastable vitreous state, crystallizes upon heating and finally melts, showing a broad endothermic peak at lower temperatures (around 50 °C) than the pure IL. This broadening of the melting peak corresponds to a heterogeneous population of crystals, which suggests the possibility of remaining polymorphism in ChCl. This melting point depression with respect to pure ionic liquid confirms the formation of a new liquid phase stabilized by specific interactions such as hydrogen bonds and ion–dipole forces.

### 2.2. Cyclic Voltammetry (CV)

**Figure 3:**
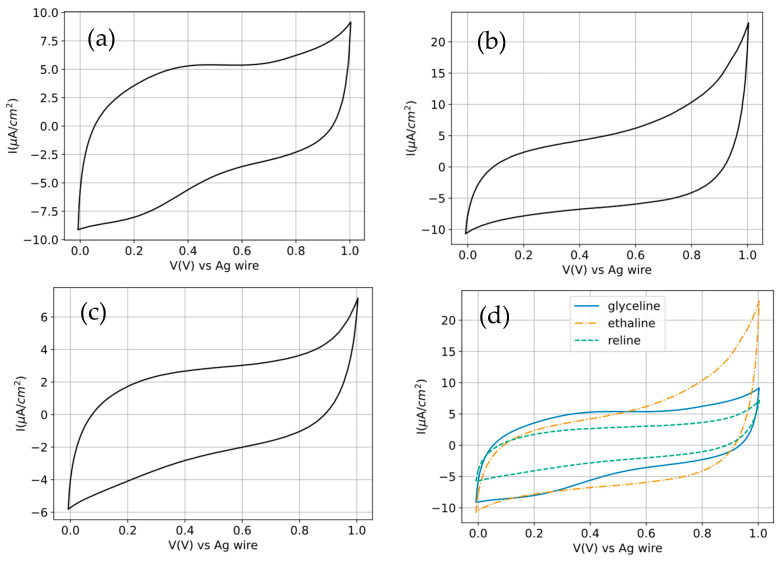
Cyclic voltammetry of (**a**) glyceline, (**b**) ethaline, and (**c**) reline and (**d**) a comparison of the three DESs. The measurements were done at a scan rate of 0.05 V s^−1^ and a temperature of 30 °C. The working electrode was glassy carbon, the counter electrode was graphite, and a silver wire was used as the pseudo-reference.

An ideal capacitor voltammogram, where only pure electrostatic charge storage occurs, would have a perfectly rectangular shape, since the current would follow the equation I=C⋅dV/dt and, as the scan rate is constant, the current would also be constant. The electrochemical behavior of the three choline chloride-based DESs (glyceline, ethaline, and reline) was evaluated by cyclic voltammetry on a glassy carbon electrode at 30 °C and at a scan rate of 0.05 V s^−1^. In all DESs, the voltammograms are largely featureless over most of the investigated potential range, with no well-defined anodic or cathodic peaks. This response indicates that the measured current is dominated by non-faradaic processes, primarily electrochemical double-layer charging at the carbon–DES interface, rather than by reversible redox reactions. Such behavior is characteristic of neat DESs in the absence of deliberately added electroactive species and has been widely reported for choline chloride-based systems on inert electrodes. Despite the qualitative similarity in voltammetric shape, clear differences in current density are observed among the three DESs. From a more detailed analysis of Figure 3, it can be observed that glyceline manifests the closest-to-ideal behavior, whereas ethaline and reline display narrower voltammograms. Nevertheless, all three DESs show quasi-rectangular voltammograms, with no significant reaction peaks being observed.

**Figure 4:**
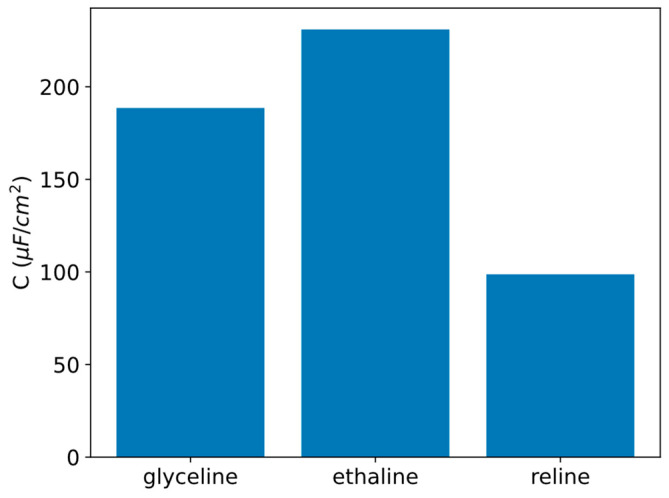
Areal capacitance of glyceline, ethaline, and reline at 30 °C.

Although ethaline presents the least rectangular shape, it also reaches the highest current intensities, making it the electrolyte with the highest capacity, as shown in Figure 4. This value is closely followed by glyceline, while reline exhibits a much lower capacitance.

The capacitance trend can be rationalized in terms of the intrinsic physicochemical properties of these solvents, particularly viscosity and ionic conductivity. Ethaline is known to possess relatively low viscosity and higher ionic conductivity compared to glyceline and reline at similar temperatures, which facilitates ion transport and results in larger capacitive currents. In contrast, glyceline is more viscous, leading to increased resistance and reduced ion mobility, while reline typically exhibits the strongest hydrogen-bonding network and the highest viscosity among the three. These factors suppress charge transport and amplify ohmic distortion, resulting in the lower current densities observed for reline. The voltammograms also display a noticeable asymmetry and tilting of the capacitive loops, particularly at higher current densities. This behavior reflects the combined effects of uncompensated solution resistance and slow interfacial reorganization in these highly structured, viscous electrolytes. Such deviations from ideal rectangular capacitive behavior are commonly observed in DESs and are consistent with their complex nanostructure and sluggish ion dynamics. At more positive potentials, all three DESs show a sharp increase in anodic current near the upper vertex of the scan. This current rise is attributed to the onset of electrolyte and/or interfacial oxidation processes, signaling an approach to the anodic stability limit of the DES–electrode system. While reline exhibits lower currents in this region, this should not be interpreted unambiguously as a wider electrochemical stability window. In viscous electrolytes, reduced current near the potential limits may also arise from kinetic and transport limitations rather than from enhanced thermodynamic stability. Moreover, the use of a silver wire pseudo-reference electrode means that absolute potential values are influenced by chloride activity and junction conditions; therefore, comparisons of stability limits are most meaningful when made under identical experimental conditions rather than directly against the literature values obtained with different reference electrodes.

Although all three DESs display predominantly quasi-rectangular voltammograms, their response deviates from the ideal rectangular shape expected for a purely electrostatic double-layer capacitor. The observed tilting, narrowing, and asymmetry of the CV loops can be attributed mainly to polarization effects arising from the relatively high ohmic resistance of the electrolyte (iR drop), together with slow ion transport and delayed interfacial reorganization in these highly structured liquids. In DESs, the strong hydrogen-bond network and high viscosity hinder ionic mobility, so the current cannot respond instantaneously to the applied potential sweep. Consequently, the voltammograms become distorted from the ideal capacitive shape. These effects are weakest for ethaline, which has the lowest resistance and highest ionic mobility, and strongest for reline, which exhibits the highest viscosity and resistive losses. Glyceline shows intermediate behavior. Therefore, the differences in voltammetric shape are consistent with the transport properties independently supported by the EIS measurements. In addition, the increase in anodic current near the upper potential limit is attributed to the onset of electrolyte and/or interfacial oxidation, indicating that the system is approaching its anodic stability limit under the present experimental conditions. Because a Ag wire pseudo-reference electrode was used, the absolute potential values should be interpreted comparatively within this study rather than directly compared with the literature values obtained using different reference electrodes [22].

The areal capacitance values provide a quantitative framework that directly rationalizes the qualitative trends observed in the cyclic voltammograms and the differences in current density among glyceline, ethaline, and reline (Figure 3).

It is worth noting that glyceline displays a voltammogram visually closer to the ideal rectangular shape than ethaline, even though its capacitance is lower. This apparent discrepancy arises because the geometric shape of the CV and the capacitance are related but not equivalent descriptors. A more rectangular profile mainly indicates that the current remains comparatively uniform during the potential sweep, whereas the capacitance depends on the total stored charge, reflected by the magnitude of the integrated current. In the present case, glyceline shows a smoother and less potential-dependent charging response, which makes its CV appear more ideal. However, ethaline sustains significantly higher charging currents and therefore yields a larger enclosed voltammetric area and a higher capacitance. This interpretation is supported by the impedance results, which show that ethaline has the lowest Rs and the highest differential capacitance, consistent with faster ion transport and more efficient interfacial packing/charging. Conversely, glyceline exhibits higher resistance and lower effective capacitance despite its more regular CV shape. Therefore, the closer-to-rectangular appearance of glyceline should be interpreted as a more uniform capacitive response, not as evidence of greater charge-storage ability. In summary, CV rectangularity should be interpreted as a descriptor of response ideality, whereas capacitance is governed by charge-storage magnitude and interfacial transport/packing efficiency.

### 2.3. Electrochemical Impedance Spectroscopy (EIS)

**Figure 5:**
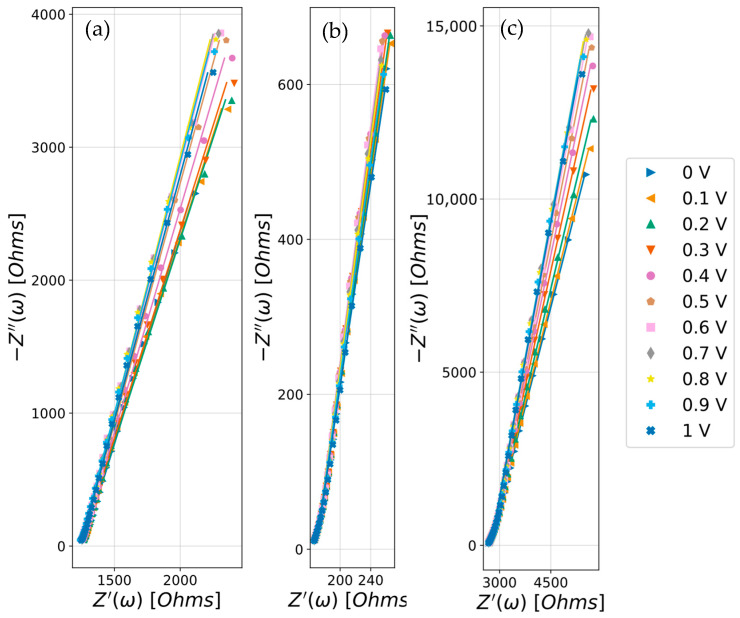
Nyquist plots of (**a**) glyceline, (**b**) ethaline and (**c**) reline at 30 °C measured with glassy carbon as the working electrode, graphite as the counter electrode and a silver wire as the pseudo-reference. Only selected data is shown. The lines represent the fitting to the R-CPE circuit.

EIS was employed to assess the interfacial charging response of the DES electrolytes and to separate double-layer contributions from purely ohmic losses. By probing the frequency-dependent impedance across the 0–1 V window, the analysis aims to provide the solution/electrolyte resistance Rs, which governs high-frequency transport limitations, and a non-ideal capacitive response associated with the electrified interface, from which a frequency-appropriate differential capacitance can be extracted. This approach is particularly relevant for viscous, highly structured ionic media such as DESs, where interfacial heterogeneity and slow structural relaxation often lead to deviations from ideal capacitive behavior. This method consisted of an alternative assessment to the areal capacitance, which was obtained by fitting impedance data to an R-CPE equivalent circuit model, as explained in the Materials and Methods Section. Figure 5 shows the Nyquist plots of the three DESs fitted to the model circuit.

While an ideal capacitor would exhibit a straight vertical line in a Nyquist plot, in our system the line appears tilted. As previously reported by Silva et al. [23], the low-frequency branch is not perfectly vertical, and the phase angle deviates from −90°, indicating a distribution of relaxation times rather than an ideal capacitor. This behavior is commonly captured with a CPE and is typically associated with surface heterogeneity/roughness, subtle faradaic processes, impurities, and/or interfacial restructuring in viscous ionic media [24].

Consequently, the impedance spectra were interpreted with a minimal *R*s-CPE framework, in which the high-frequency real-axis intercept directly yields the electrolyte (ohmic) Rs and enables a straightforward comparison of transport properties among the DESs.

**Figure 6:**
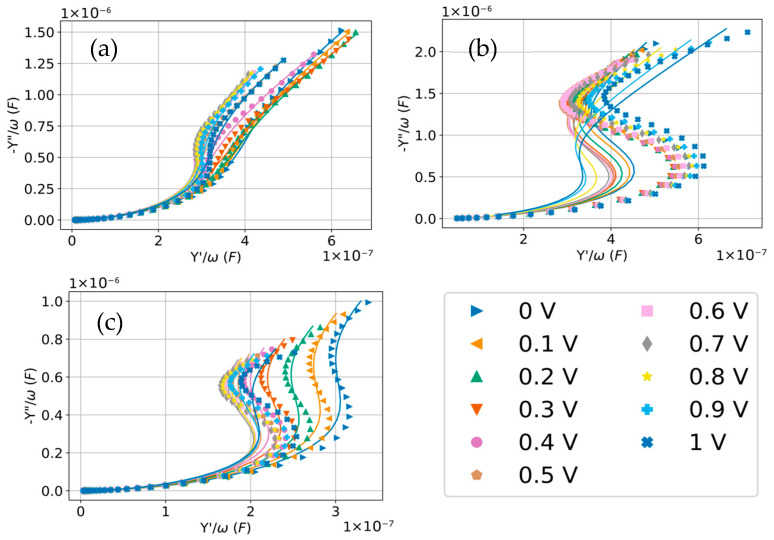
Y″/ω vs. Y′/ω plots of (**a**) glyceline, (**b**) ethaline and (**c**) reline showing the quality of the fit to the complete range of frequencies.

Accordingly, at 0.5 V, the Rs extracted from the R-CPE fitting was 168.60 ± 0.56 Ω for ethaline, 1246.2 ± 3.4 Ω for glyceline, and 2734.0 ± 8.9 Ω for reline, confirming substantially faster charge transport in ethaline (Table A1, Table A2 and Table A3 in the Appendix A). Moreover, according to the relation Z = 1/jωC, DESs that exhibit higher capacity are those reaching lower values of imaginary impedance (Z″). Therefore, the highest capacity corresponds to ethaline, followed by glyceline and reline, in agreement with Figure 4. As discussed, Figure 5 is very representative of the electrical behavior of the samples. However, as noted by Silva et al. [23], deviations from the model can be more easily detected in Y″/ω vs. Y′/ω plots, shown in Figure 6.

Figure 6 presents the frequency-normalized admittance response after subtraction of the series resistance, plotted as Y″/ω versus Y′/ω. This representation is closely related to the complex capacitance and is widely used because it emphasizes interfacial charging while de-emphasizing purely ohmic contributions. In the complex-capacitance plane, a single ideal double-layer time constant collapses to a point, whereas interfacial charging appears as a line or arc, often well approximated by a constant phase element (CPE).

Figure 6 shows the clear deviations from the model in the low-frequency range. In all three electrolytes, the intermediate-to-high-frequency portions of the curves follow the CPE-type trend expected for non-ideal double-layer charging, consistent with a distribution of relaxation times rather than a single Debye-like capacitor. Such dispersion is a common hallmark of concentrated ionic media, where interfacial heterogeneity and microscopic configurational dynamics (ion packing and local rearrangements) broaden the relaxation spectrum and produce phase angles that deviate from 90° even in nominally blocking conditions [25]. The key outcome of Figure 6, however, is the systematic low-frequency deviation of the experimental points from the single R-CPE fit, most evident as curvature and loop-like features that cannot be reproduced by a one-process charging element. In the complex-capacitance/admittance-plane interpretation for ionic liquids, this behavior is most naturally assigned to an additional slow interfacial process that becomes resolved only at long times: potential-dependent reorganization of the interfacial ionic layers, slow adsorption restructuring of specifically interacting species, and/or weak faradaic leakage pathways that introduce an extra relaxation. These slow dynamics have been repeatedly documented for ionic liquid/electrode interfaces using combined structural probes (STM/AFM) and impedance analysis, and they are also anticipated on general grounds because the structural relaxation of dense ionic interfacial layers can be intrinsically slow and strongly bias-dependent. In this context, the increasing misfit observed as one progresses from ethaline to glyceline and particularly to reline can be rationalized by the progressively slower bulk and interfacial dynamics of the more viscous DESs: the strong hydrogen-bond network imposes constraints on ion diffusion reorientation, which shift slow interfacial relaxations to lower characteristic frequencies and broaden the frequency interval over which a simplified model may fail, in particular for an extended electrochemical window (0–1 V vs. Ag (wire)). This trend is consistent with independent rheological/dynamical studies comparing these canonical DESs, which show markedly different relaxation/transport time scales across glyceline, ethaline, and reline [26]. The viscosities of the DESs reported in the literature, presented in Table 1, provide a consistent macroscopic descriptor of this fact, supporting this interpretation. As DESs are significantly more viscous than aqueous electrolytes, diffusion-controlled processes become rate-limiting, particularly in the low-frequency regime. The correlation between viscosity and non-ideal capacitive response supports a mechanistic picture in which hindered ionic transport, surface adsorption, and interfacial restructuring contribute collectively to the observed impedance behavior.

**Figure 7:**
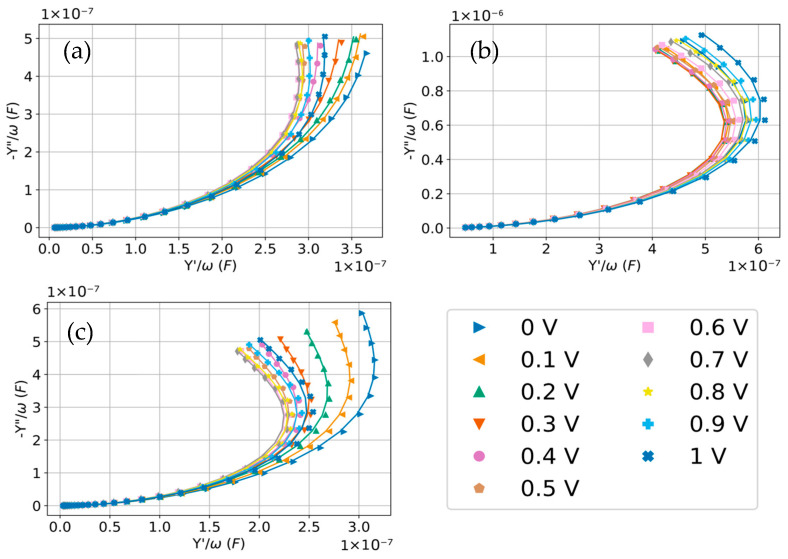
Y”/ω vs. Y’/ω plots of (**a**) glyceline, (**b**) ethaline and (**c**) reline showing the quality of the fit in the selected range of frequencies.

Accordingly, to isolate the interfacial charging regime that is adequately described by the R-CPE model and to minimize contributions from slow low-frequency processes, the analysis was repeated after excluding the data points in the lowest-frequency range. Figure 7 shows the Y″/ω vs. Y′/ω plots of the three DESs after discarding the low-frequency points, resulting in a much cleaner fit. Figure 7 also highlights that, once the lowest-frequency points are excluded, the remaining data are well described by a simple R-CPE equivalent circuit. This supports the fact that, over the selected frequency window, the measured response is dominated by one main interfacial charging process that behaves in a quasi-capacitive manner but non-ideally (i.e., with a distributed relaxation). In contrast, the low-frequency deviations seen previously most naturally indicate that an additional slow contribution becomes important at long times and is not captured by the minimal circuit. A low-frequency structure can reflect real, slow interfacial dynamics (reorganization, adsorption-related relaxations, or faradaic process), and whether those are considered part of the double layer depends on the level of description that is adopted.

To ensure that the analysis reflected predominantly an electrical double-layer structure rather than contributions from faradaic reactions, the structure was considered a conservative blocking criterion in a previous work dedicated to EDL DES/electrode study. Specifically, Costa et al. [12] defined a cutoff current density below 10 µA cm^−2^ and treated measurements as representative of the double-layer regime only when the current remained below this threshold in both cyclic voltammetry and impedance experiments. Operationally, this criterion served two purposes. In CV, it screens out potential regions where redox reactions, impurity-driven processes, or electrode surface chemistry produce measurable charge-transfer currents, restricting the analysis to current densities (<10 µA cm^−2^), and therefore emphasizes the non-faradaic, capacitive component of the response. In EIS, the same cutoff provides a consistency check that the applied potential lies in a regime where the interface behaves close to an ideal capacitor, so that the measured impedance is dominated by interfacial charging rather than by charge-transfer resistance and associated faradaic kinetics. Applying a shared criterion across CV and EIS strengthens the internal coherence of the dataset: capacitance values extracted from EIS can be discussed in the same potential window that appears non-faradaic in CV, reducing the risk that differences among electrolytes are driven by parasitic redox activity rather than genuine changes in interfacial structure and dynamics.

**Figure 8:**
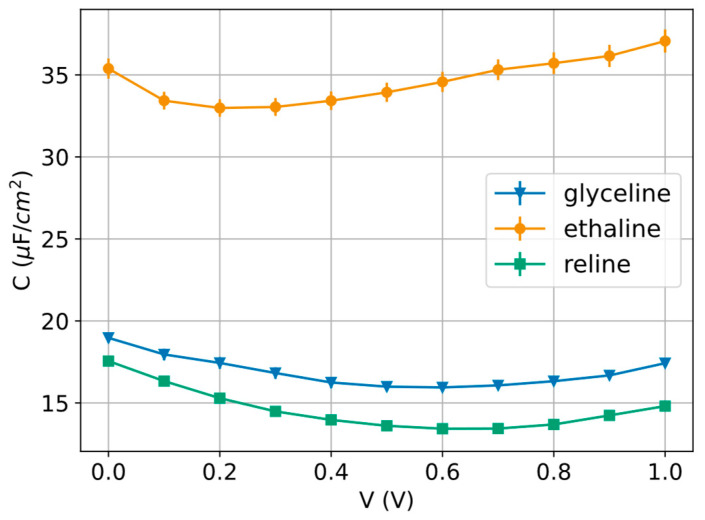
Differential capacity curves of glyceline, ethaline and reline at 30 °C. In this case, the lines between data points serve merely as visual aids and do not represent a fit.

The differential capacitance obtained from fitting the EIS data over the appropriate frequency range is plotted as a function of potential in Figure 8.

The three C(E) curves exhibit the same overall shape: a shallow, U-shaped dependence of differential capacitance on applied potential, with a broad minimum at intermediate potential and higher capacitance toward both ends of the explored window (0–1 V). This is the classical signature of interfacial charging in an electrical double layer: as the electrode is driven away from the potential where the net interfacial charge is smallest, the surface charge density increases, and the incremental (differential) capacitance rises accordingly. In this sense, the position of the minimum is best interpreted as an operational capacitance minimum potential (*E* min) rather than a definitive PZC, because capacitance minimum and true zero-charge conditions can be offset in concentrated, structurally complex electrolytes.

Across the full potential range, ethaline shows the highest capacitance, approximately 33–37 μF cm^−2^, with a minimum near ∼0.2/0.3 V and a steady increase toward 1 V. Glyceline is intermediate, roughly 16–19 μF cm^−2^, with a minimum around ∼0.5/0.6 V and only a modest upturn at higher potentials. Reline shows the lowest values, about 13–18 μF cm^−2^, with its minimum also near ∼0.5/0.6 V and a clearer increase toward 1 V. The consistent ordering *C* ethaline > *C* glyceline > *C* reline indicates that, under identical electrode geometry and temperature, the DES chemistry controls the effective compactness of the charge-separation zone: higher *C* implies a smaller effective charge-separation distance and/or a larger effective permittivity and accessible ion density at the interface.

Interpreted physically, the higher capacitance of ethaline is consistent with a more efficient interfacial packing/charging response (often associated with higher ionic mobility and a thinner effective interfacial layer), whereas the lower capacitance of reline is consistent with a more sluggish and/or more strongly associated interfacial structure (which can increase the effective thickness of the charge-separation region and reduce the incremental charge stored per unit potential). The fact that glyceline and reline minima occur at similar potentials, while ethaline’s minimum is shifted to a lower potential, also suggests that the balance between cation- and anion-dominated interfacial layering and any weak specific interactions differs among the three DESs (or interactions are preferred in the form of HBD–anion or HBD–cation). Finally, the curvature is relatively smooth (especially for glyceline), which is typical when the interface is strongly structured, and the capacitance reflects a broadened spectrum of interfacial configurations rather than a sharp, ideally polarizable double layer dominated by neutral HBD-free fraction (organic-like shape).

### 2.4. Nuclear Magnetic Resonance (NMR)

**Figure 9:**
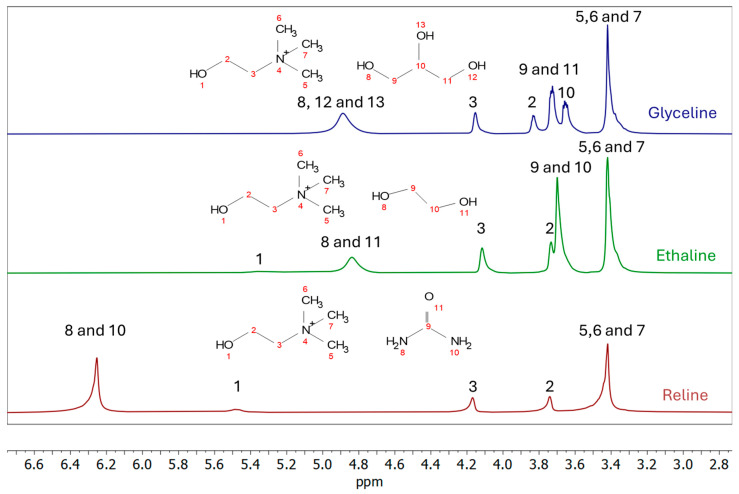
^1^H NMR spectra of glyceline, ethaline and reline at 30 °C. The tagged numbers identify the hydrogen atoms.

The chemical shift in the ^1^H NMR spectra for the three DESs studied is summarized in Table 2. For the three DESs, the CH_3_ signal of choline cation used as a reference, according to the results obtained by Abbott et. al. [9], to enable direct comparison among the DES spectra, presented in Figure 9. For reline, the recorded spectra are coincident with the results obtained by Abbott et. al. [9], indicating the homogeneity of the mixture without evidence of side reactions or covalent bonds, which is expected for properly synthesized DES. Similarly, the spectrum of ethaline agrees with that published by AlZaharani et al. [26]. Again, for this system, the characteristic peaks of choline cation and ethylene glycol are still present in the spectra. This is an indicator of the presence of these compounds without side reactions when mixing them, obtaining the desired DES.

As noted above, in general, all studied DESs exhibit the resonances of the choline cation, particularly the N^+^(CH_3_)_3_ group, as well as the CH_2_–OH and CH_2_–N^+^ protons (see Table 2). The broadening observed for the OH and NH signals is consistent with fast proton exchange due to the labile nature of these protons. No significant shifts were observed for the choline cation when the CH_3_ group was referenced at 3.42 ppm for all DESs. For the CH_2_–OH protons (labeled as 2 in Figure 9), the chemical shifts were 3.74, 3.73, and 3.83 ppm for reline, ethaline, and glyceline, respectively. The CH_2_–N^+^ protons (labeled as 3) appeared at 4.16, 4.11, and 4.15 ppm for reline, ethaline, and glyceline, respectively. The slightly downfield shift observed for glyceline suggests stronger hydrogen-bonding interactions in this system compared to the other DESs.

Overall, the results are consistent with the formation of supramolecular structures mediated by hydrogen bonding in all three systems. No evidence of chemical reaction between the mixed compounds is observed, supporting the formation of the corresponding DESs.

## 3. Discussion

DSC results show that the addition of solvents causes an effective destabilization of the crystalline network of the ChCl, leading to the derangement of its melting point and the inhibition of crystallization mainly for glyceline and reline. Depending on the solvent, the system evolves into a completely amorphous compound or, as in the case of ethaline, it forms, especially at low temperatures, a hybrid morphology comprising crystalline regions embedded in an amorphous environment. This behavior confirms that mixtures are not simple solutions but new liquid systems with clearly differentiated thermal properties, consistent with the formation of deep eutectic solvents.

Beyond the qualitative classification of the voltammograms as quasi-rectangular, the differences among the three DESs suggest distinct balances between interfacial charge accommodation and bulk ion rearrangement. In an ideally polarizable electrolyte, the current response follows the potential sweep nearly instantaneously and remains almost constant; here, however, the progressive distortion from glyceline to ethaline and especially to reline indicates that the charging process is increasingly governed by finite relaxation times within the liquid. In DESs, these relaxation times are not determined solely by ion diffusion in the usual dilute-electrolyte sense, but also by the reorganization of a strongly correlated hydrogen-bonded matrix in which choline, chloride, and HBD molecules are dynamically coupled [28,29,30]. Thus, the voltammetric shape reflects not only capacitance, but also how rapidly the local interfacial structure can adapt to the changing electrode polarization. From this perspective, ethaline appears to benefit from a more labile interfacial environment, capable of sustaining higher charging currents despite stronger geometric distortion of the CV loop, while reline behaves as the most dynamically constrained system, where slow structural rearrangement suppresses current response over the whole scan [31]. Glyceline occupies an intermediate regime in which the interface responds more uniformly with potential, giving a visually more rectangular profile, but without matching the higher charge-storage efficiency of ethaline. This distinction is important because it shows that voltammetric “ideality” and charge-storage magnitude are not equivalent descriptors in highly structured electrolytes. The Nyquist plots indicate that the electrochemical response cannot be reduced to a simple separation between a pure series resistance and an ideal double-layer capacitor. The depressed, tilted low-frequency branch reveals that the electrified interface behaves as a spatially and dynamically heterogeneous system with a distribution of charging times. In physical terms, this means that different microscopic regions of the interface do not charge synchronously: some domains respond relatively fast, whereas others require slower ion displacement, hydrogen-bond rearrangement, or local restructuring of ion–HBD aggregates before reaching the same degree of polarization [29]. This interpretation is particularly relevant for DESs, where the interfacial region is expected to be compositionally stratified and structurally anisotropic [30,32]. Accordingly, the CPE behavior should not be viewed merely as an empirical fitting choice but as a signature of a broadened spectrum of interfacial relaxation modes [33]. The much lower Rs of ethaline indicates that its bulk phase imposes the smallest transport penalty, but the impedance response further suggests that its interface is also more efficient at redistributing charge once polarization is applied. In contrast, the larger resistance and stronger non-ideality in glyceline and especially reline imply that the compact layer is formed under progressively stronger kinetic constraints, so that the observed capacitance becomes increasingly limited by the ability of the interfacial network to reorganize rather than by simple ion availability alone. The −Y″/ω versus Y′/ω representation provides a more revealing view of the interfacial dynamics because it effectively filters out the dominant ohmic contribution and emphasizes how the capacitance is distributed across time scales. In this representation, the deviation from the single R-CPE model at low frequencies is especially significant: it shows that, once sufficient time is allowed for charging, the interface does not simply approach an idealized steady double-layer but instead begins to express additional slow modes that are hidden at higher frequencies. These modes are consistent with potential-dependent restructuring of the adsorbed ionic layers, slow exchange between differently coordinated ionic species, and reorientation of the hydrogen-bond network near the electrode surface. In other words, the low-frequency departure from the fit is not merely a fitting imperfection; it is evidence that the electrified DES/electrode interface has internal structural dynamics of its own. The fact that these deviations intensify from ethaline to glyceline to reline strongly suggests that the characteristic time for interfacial equilibration becomes progressively longer across this series. This trend supports a mechanistic picture in which the better performance of ethaline arises not only from lower bulk viscosity but also from a less constrained interfacial architecture that can reorganize more efficiently under polarization. Conversely, in reline, the stronger association and slower restructuring appear to broaden the relaxation spectrum so substantially that the single-process description fails earlier and more severely, particularly in the long-time regime.

Taken together, the CV, Nyquist, and admittance-plane analyses indicate that the interfacial response of these DESs is controlled by an interplay between bulk ion transport and slow structural adaptation of the electrical double layer. This point is essential for interpreting the differential capacitance curves. In concentrated hydrogen-bonded electrolytes, the measured capacitance is not determined solely by the number of ions available to screen the electrode but also by how efficiently those ions and associated HBD-rich entities can reorganize into a compact and polarizable interfacial structure. Therefore, the higher differential capacitance of ethaline should be interpreted as the signature of a more responsive and compact charge-separation region, whereas the lower values for glyceline and reline reflect increasingly hindered interfacial packing and slower configurational equilibration under applied potential [34].

A more quantitative correlation can be established between the hydrogen-bond network and the electrochemical response of the three DESs. The 1H NMR data show that the choline CH_2_ OH resonance is located at 3.73 ppm in ethaline, 3.74 ppm in reline, and 3.83 ppm in glyceline, with the downfield shift in glyceline indicating stronger local hydrogen-bonding interactions around that site. However, the bulk rigidity of the liquid is more clearly reflected by the transport properties. At 30 °C, the viscosity increases markedly from ~45 mPa·s for ethaline to 264–267 mPa·s for glyceline and 1028.3 mPa·s for reline. In parallel, the representative literature values of ionic conductivity at room temperature decrease from ~7.6–8.3 mS cm^−1^ for ethaline [35,36,37] to ~1.47 mS cm^−1^ for glyceline [18] and ~0.6–1.3 mS cm^−1^ for reline [16,38]. This transport hierarchy is fully consistent with the EIS results, where the fitted solution resistance at 0.5 V rises from 168.60 Ω for ethaline to 1246.2 Ω for glyceline and 2734.0 Ω for reline. The differential capacitance follows the opposite trend, with ethaline showing the highest values (~33–37 μF cm^−2^), glyceline intermediate values (~16–19 μF cm^−2^), and reline the lowest values (~13–18 μF cm^−2^). Taken together, these results indicate that the more weakly constrained network in ethaline enables faster ionic rearrangement, lower ohmic losses, and more efficient interfacial charge storage. In contrast, the more strongly associated and dynamically slower glyceline and especially reline exhibit progressively hindered ion transport, higher Rs, and lower capacitance. Importantly, the 1NMR shift reflects local hydrogen-bonding around a specific molecular environment, whereas viscosity, conductivity, and Rs probe the collective dynamics of the entire supramolecular network; therefore, local hydrogen-bond strength and bulk transport limitations should be regarded as complementary, rather than identical, descriptors. In conclusion, moving from ethaline to glyceline to reline, the increase in viscosity (~1:6:23 relative to ethaline) is accompanied by an increase in Rs (~1:7:16) and a decrease in differential capacitance to roughly one-half of the ethaline value.

Although the electrochemical characterization in the present work was carried out at 30 °C, the broader temperature feasibility of ChCl-based DES electrolytes is supported by both our thermal analysis and published interfacial electrochemistry data [39]. First, the DSC results obtained here show that glyceline, ethaline, and reline remain liquid well below room temperature, indicating favorable low-temperature phase stability. In addition, Costa et al. [12] reported temperature-dependent CV and differential capacitance measurements for related ChCl-based DESs, including ethaline and reline over 30–60 °C and diol-based analogs up to 70 °C, on GC, Au, and Pt electrodes. The results showed that increasing temperature generally raises the capacitive current response, consistent with improved ion mobility and lower transport limitation, but also tends to decrease the ideal polarizability window. For GC, the differential capacitance increased systematically with temperature for all the DESs studied, whereas for Au and Pt, the temperature coefficient depended on both the electrode material and the HBD identity [40]. Therefore, the available evidence indicates that these DES electrolytes are feasible over a broad temperature interval, but their electrochemical performance reflects a trade-off between enhanced transport at higher temperatures and some narrowing of the purely capacitive stability window.

## 4. Materials and Methods

### 4.1. Chemicals

Three DESs based on choline chloride were prepared in a molar proportion of 2 parts of HBD for one part of ChCl. Studied proportions of ChCl with the different HBDs were selected according to the previous literature [10,20,41]. Precursors were maintained under vacuum for at least 24 h. The selected combinations have been studied before and have common names. The DES based on the combination of ChCl with glycerol is named glyceline, whereas that based on ethylene glycol is denominated ethaline, and when ChCl is combined with urea, it is called reline. Table 3 summarizes the name, molecular mass, chemical structure, CAS number, purity, and provenance of the chemicals used in this work.

### 4.2. Experimental Techniques

#### 4.2.1. Differential Scanning Calorimetry (DSC)

Thermal transitions of the DES and ChCl were determined with a DSC Q2000 (Waters-TA-Instruments, New Castle, DE, USA). Two preliminary thermal ramps were applied to all samples to ensure the quality of the results:

(1) Heating from 40 °C to 125 °C at 40 °C/min;
(2) Isothermal step at 125 °C for 45 min to remove impurities that can affect the thermal behavior, such as free water, and to erase the thermal history of the sample.

Then, the following heating and cooling ramps were performed:

(3) Cooling to −80 °C at 5 °C/min;
(4) Heating to 100 °C at 5 °C/min;
(5) Cooling to −80 °C at 10 °C/min;
(6) Heating to 100 °C at 10 °C/min.

Isothermal steps of 30 s were introduced between heating and cooling ramps to ensure temperature stabilization.

All experiments were performed under a nitrogen atmosphere (50 mL/min) with a mass sample between (7 and 10) mg. Sealed aluminum pans (50 μL) with a hole that allows water to escape when it evaporates were used. All the experiments were repeated at least twice to ensure results. Temperature and heat (enthalpy) calibrations were performed using the melting temperature of indium and zinc and the melting heat (enthalpy) of indium, respectively. Figures were generated using Seaborn v0.13.2 [42] with a colorblind-friendly palette to ensure accessibility. This visualization approach was applied consistently across most of the plots presented in this work.

#### 4.2.2. Cyclic Voltammetry 

Cyclic voltammetry experiments were carried out using an AUTOLAB PGSTAT302N potentiostat (Metrohm, Herisau Switzerland) in a three-electrode configuration consisting of a glassy carbon working electrode, a graphite rod counter electrode, and a silver wire as pseudo-reference. Prior to each measurement, nitrogen was bubbled through the solution to remove any dissolved oxygen. During the experiments, a constant flow of N_2_ was maintained to prevent oxygen-induced redox reactions and to minimize moisture absorption from the air. Measurements were performed between 0 and 1 V at a scan rate of 0.05 V/s.

The areas of the voltammograms were integrated to obtain the areal capacitance, according to

$$C=\frac{1}{\nu A\Delta V}\int IdV$$

where C is the areal capacitance, ν the scan rate, A the area of the working electrode, ΔV the potential range, I the current, and V the potential at the working electrode [43]. All electrochemical potentials reported in this work are referenced to a silver wire pseudo-reference electrode. Since the potential depends on the composition of the chloride-containing DES and associated junction conditions, the reported values should be interpreted on an internal comparative scale and not directly converted to Ag/AgCl, RHE, or SHE in the absence of an independent calibration. The CV measurements shown in this work were recorded at a single scan rate (0.05 V s^−1^) and are used here mainly as a comparative electrochemical screening of the three DESs under identical experimental conditions. Although the voltammograms display a largely quasi-rectangular profile and no pronounced redox peaks, a more rigorous confirmation of the capacitive nature of the response would require measurements at multiple scan rates. It should also be noted that no iR compensation was applied; thus, the observed distortion from ideal rectangular behavior includes the effect of uncompensated solution resistance, particularly in the more resistive DES media. Accordingly, the voltammetric response is discussed together with the EIS results, which offer a more quantitative analysis of the resistive and capacitive contributions. Measurements were taken by approaching the target temperature from higher values to avoid crystallization.

#### 4.2.3. Water Content

Karl Fischer titration was performed for all samples using a Karl Fischer Coulometer (Utrecht, The Netherlands), with a drift stop criterion of <20 µL min^−1^. The water content measured after the electrochemical experiments ranged from 0.8 to 1.2 *w*/*w*%, which is consistent with the values reported by Nolasco et al. [44] for DES prepared by drying the individual components and weighing them inside a glovebox in sealed glass vials. For comparison, dried DES was prepared and stored under high vacuum (ca. 10−1 Pa) in a Schlenk flask at room temperature for at least four days. Although this procedure yielded slightly lower water contents (0.5–0.7 *w*/*w*%), the use of molecular sieves in the electrochemical cell during the measurements proved to be an effective approach to minimizing moisture interference, representing a practical alternative to high-vacuum treatment [12].

#### 4.2.4. Electrochemical Impedance Spectroscopy (EIS)

Electrochemical impedance spectroscopy (EIS) measurements were conducted using the same three-electrode configuration employed for cyclic voltammetry. Each sample was analyzed at potentials from 0 to 1 V in 0.1 V increments. The impedance spectra were recorded over a frequency range of 2 Hz to 20 kHz. Capacity values were obtained following the methodology described in [23]. The EIS data were fitted to an equivalent circuit consisting of a resistance in series with a constant phase element (CPE). After fitting, Y″/ω vs. Y′/ω plots (with Y denoting the admittance and ω the frequency) were used to refine the frequency range. Low-frequency data, where clear deviations from the fitting were observed, were discarded and the fitting was repeated. Following the publications of Silva et al. [23], capacity was obtained from the relation $C=Y_{0}^{1/n}R^{\left(1-n\right)/n}$, where $Y_{0}$ and n are the parameters of the admittance of the CPE, which is of the form $Y_{CPE}=Y_{0}{\left(j\omega \right)}^{n}$. Fittings and errors were obtained using the Python packages impedance.py v1.7.1 [45] and uncertainties.py v3.2.3 [46].

#### 4.2.5. Nuclear Magnetic Resonance (NMR)

The 1H NMR spectra were obtained using a Bruker Neo 750 spectrometer with a magnetic field of 17.6 T and a proton resonance frequency of 750 MHz, without sample rotation. The 1D 1H spectrum was obtained under quantitative conditions using a low excitation tilt pulse angle of 5 degrees, 64 scans with an inter-scan delay (d1) of 4 s and an acquisition time (aq) of 2.88 s. The samples were prepared in 5 mm standard tubes and stored at 298 K to perform the measurement.

## 5. Conclusions

This study provides a comprehensive evaluation of three deep eutectic solvents, from mixtures of the ionic liquid choline chloride and glycerol, ethylene glycol, and urea, as potential electrolytes for supercapacitors, allowing for several key conclusions to be drawn.

Differential scanning calorimetry experiments show that both glyceline and reline exhibit predominantly amorphous behavior, as evidenced by the absence of crystallization or melting peaks in the whole studied temperature interval. In contrast, ethaline seems to display a combination of both amorphous and ordered phases. Overall, it is satisfactory to note that all three HBDs selected can maintain the resulting DES in a liquid state even well below room temperature.

Electrochemical characterization reveals that the three DESs exhibit quasi-rectangular cyclic voltammograms. Among them, ethaline shows the highest capacitance, followed by glyceline and reline. Electrochemical impedance spectroscopy measurements corroborate this trend and additionally indicate only a modest dependence of capacity on potential, with the differential capacitance curve presenting the characteristic U-shape predicted by GCS theory. Most importantly, ethaline displays the lowest resistance among the electrolytes studied, followed again by glyceline and lastly reline, highlighting its superior charge transport properties within the studied mixtures.

^1^H NMR analysis of the three studied DESs confirms the successful formation of the intended mixtures without evidence of side reactions or covalent bond formation. All spectra display the characteristic resonances of the choline cation, with chemical shifts consistent with the literature values. Minor downfield shifts were observed in glyceline, indicating stronger hydrogen-bonding interactions in this system compared to reline and ethaline. Overall, the NMR results validate the structural integrity and homogeneity of the synthesized DESs, while highlighting subtle differences in hydrogen-bonding environments that reflect the nature of the hydrogen bond donors in each system.

The results presented in this study open interesting paths for future research. Related to the thermal analysis area, it would be valuable to perform experiments using different heating and cooling rates on samples with identical thermal histories. Also, repeating the electrochemical experiments on full supercapacitor devices would be essential to determine the behavior and suitability of these candidate electrolytes with different electrode materials. Finally, evaluating long-term stability and cycling performance would be crucial to determine the viability of these DESs as electrolytes for supercapacitors.

The results presented in this study open several relevant directions for future research. From a fundamental perspective, further systematic investigation of physicochemical properties would be necessary to complete the understanding of the behavior of these systems. In the thermal analysis field, for instance, performing experiments with different heating and cooling rates on samples with identical thermal histories would provide deeper insight into their phase behavior. Additionally, exploring the effect of varying HBD molar ratios could help establish more precise structure–property–performance relationships.

From an applied standpoint, validating these DESs in real full supercapacitor devices is essential to assess their practical suitability. This includes testing their performance with different electrode materials, as well as evaluating cycling behavior under realistic operating conditions. Addressing both the fundamental characterization and the practical implementation will be crucial to fully determine the viability of these compounds as electrolytes for supercapacitors.

## Figures and Tables

**Table 1:** Dynamic viscosities of the studied DESs at 30 °C.

DES Name	Viscosity (mPa s)
Glyceline	264–267 [18]
Ethaline	~45 [19]
Reline	1028 [27]

**Table 2:** Peak shift in ppm for the DESs studied. S: singlet; M: multiplet. The tagged numbers to identify the hydrogen atoms are the same as those used in Figure 9.

0BReline						
1BIdentification	2B5, 6 and 7 (CH_3_)	3B2 (CH_2_)	4B3 (CH_2_)	5B1 (OH)	6B8 and 10 (NH_2_)	
7BPeak shift (ppm)	8B3.42 (S)	9B3.74 (S)	10B4.16 (S)	11B5.47 (S)	12B6.25 (S)	
**13BEthaline**						
14BIdentification	15B5, 6 and 7 (CH_3_)	16B9 and 10 (CH_2_)	17B2 (CH_2_)	18B3 (CH_2_)	19B8 and 11 (OH)	20B1 (OH)
21BPeak shift (ppm)	22B3.42 (S)	23B3.7 (S)	24B3.73 (S)	25B4.11 (S)	26B4.83 (S)	27B5.31 (S)
**28BGlyceline**						
29BIdentification	30B5, 6 and 7 (CH_3_)	31B10 (CH)	32B9 and 11 (CH_2_)	33B2 (CH_2_)	34B3 (CH_2_)	35B8, 12 and 13 (OH)
36BPeak shift (ppm)	37B3.42 (S)	38B3.65 (M)	39B3.72 (M)	40B3.83 (M)	41B4.15 (S)	42B4.89 (S)

**Table 3:** Chemical specifications of the reagents used.

Name/ CAS Number	Molecular Mass (g·mol^−1^)	Structure	Short/DES Name	Purity Provenance
Choline chloride 67-48-1	139.62	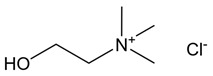	ChCl	>98% Iolitec (Heilbronn, Germany)
Glycerol 56-81-5	92.09	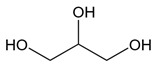	Glyceline	>99% Merck (Darmstadt, Germany)
Ethylene glycol 107-21-1	62.07	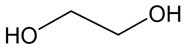	Ethaline	>99% Merck (Darmstadt, Germany)
Urea 57-13-6	60.06	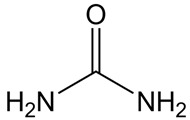	Reline	99.5% Merck (Darmstadt, Germany)

## Data Availability

Most of the data supporting the findings of this study are included within the article. Further information is available upon request.

## Abbreviations

The following abbreviations are used in this manuscript:

CPE	Constant Phase Element
CV	Cyclic Voltammetry
ChCl	Choline Chloride
DES	Deep Eutectic Solvent
EIS	Electrochemical Impedance Spectroscopy
GCS	Gouy–Chapman–Stern
HBA	Hydrogen Bond Acceptor
HBD	Hydrogen Bond Donor
IL	Ionic Liquid
PZC	Potential of Zero Charge
RTIL	Room-Temperature Ionic Liquid
NMR	Nuclear Magnetic Resonance

## Appendix A

The numbers in parenthesis indicate the uncertainties in the last two significant digits of the reported values. For example, 2.381(20) $\cdot \;10^{-6}$ means that the measured value is 2.381 $\cdot \;10^{-6}$ and its absolute uncertainty is 0.020 $\cdot \;10^{-6}$.

**Table A1:** Fit parameters of impedance data to the R-CPE model for glyceline.

V (V)	R (Ω)	$Y_{0}\;\left(\Omega^{-1}s^{n}\right)$	n
0.0	1260.9(2.1)	$2.381(20)\;\cdot \;10^{-6}$	0.8074(13)
0.1	1246.3(3.4)	$2.405(25)\;\cdot \;10^{-6}$	0.8003(16)
0.2	1245.2(3.6)	$2.380(26)\;\cdot \;10^{-6}$	0.7986(17)
0.3	1242.9(3.8)	$2.227(25)\;\cdot \;10^{-6}$	0.8037(17)
0.4	1246.4(4.0)	$1.975(22)\;\cdot \;10^{-6}$	0.8161(17)
0.5	1246.2(3.4)	$1.782(17)\;\cdot \;10^{-6}$	0.8284(15)
0.6	1241.3(3.0)	$1.694(14)\;\cdot \;10^{-6}$	0.8350(13)
0.7	1245.0(2.7)	$1.674(12)\;\cdot \;10^{-6}$	0.8374(11)
0.8	1242.8(2.9)	$1.677(14)\;\cdot \;10^{-6}$	0.8390(13)
0.9	1236.1(2.7)	$1.755(14)\;\cdot \;10^{-6}$	0.8354(12)
1.0	1243.3(2.8)	$1.873(15)\;\cdot \;10^{-6}$	0.8313(13)

**Table A2:** Fit parameters of impedance data to the R-CPE model for ethaline.

V (V)	R (Ω)	$Y_{0}\;\left(\Omega^{-1}s^{n}\right)$	n
0.0	168.29(53)	$2.484(26)\;\cdot \;10^{-6}$	0.9063(14)
0.1	168.21(52)	$2.361(23)\;\cdot \;10^{-6}$	0.9062(13)
0.2	168.29(53)	$2.294(22)\;\cdot \;10^{-6}$	0.9082(13)
0.3	168.46(54)	$2.225(22)\;\cdot \;10^{-6}$	0.9119(13)
0.4	168.54(56)	$2.192(22)\;\cdot \;10^{-6}$	0.9148(13)
0.5	168.60(56)	$2.186(23)\;\cdot \;10^{-6}$	0.9167(14)
0.6	168.56(57)	$2.211(24)\;\cdot \;10^{-6}$	0.9174(14)
0.7	168.38(56)	$2.293(25)\;\cdot \;10^{-6}$	0.9154(14)
0.8	168.31(57)	$2.348(26)\;\cdot \;10^{-6}$	0.9139(15)
0.9	168.12(57)	$2.462(28)\;\cdot \;10^{-6}$	0.9096(15)
1.0	167.94(56)	$2.655(30)\;\cdot \;10^{-6}$	0.9035(15)

**Table A3:** Fit parameters of impedance data to the R-CPE model for reline.

V (V)	R (Ω)	$Y_{0}\;\left(\Omega^{-1}s^{n}\right)$	n
0.0	2715.8(6.7)	$1.5773(96)\;\cdot \;10^{-6}$	0.8384(11)
0.1	2716.0(6.6)	$1.4456(82)\;\cdot \;10^{-6}$	0.8425(10)
0.2	2717.8(7.4)	$1.3030(77)\;\cdot \;10^{-6}$	0.8498(11)
0.3	2723.7(8.0)	$1.1639(71)\;\cdot \;10^{-6}$	0.8597(11)
0.4	2729.3(8.4)	$1.0707(66)\;\cdot \;10^{-6}$	0.8674(11)
0.5	2734.0(8.9)	$1.0031(63)\cdot \;10^{-6}$	0.8737(12)
0.6	2736.7(9.2)	$9.610(62)\;\cdot \;10^{-6}$	0.8783(12)
0.7	2739.5(9.6)	$9.412(63)\;\cdot \;10^{-7}$	0.8815(12)
0.8	2740.1(9.8)	$9.476(66)\;\cdot \;10^{-7}$	0.8828(13)
0.9	2741(10)	$9.827(74)\;\cdot \;10^{-7}$	0.8826(14)
1.0	2739(11)	1.0282(85) · 10^−6^	0.8810(15)

## References

[1] Quintana A.A., Sztapka A.M., de Carvalho Santos Ebinuma V., Agatemor C. (2022). Enabling Sustainable Chemistry with Ionic Liquids and Deep Eutectic Solvents: A Fad or the Future?. Angew. Chem.-Int. Ed..

[2] de Jesus S.S., Maciel Filho R. (2022). Are Ionic Liquids Eco-Friendly?. Renew. Sustain. Energy Rev..

[3] Radošević K., Cvjetko Bubalo M., Gaurina Srček V., Grgas D., Landeka Dragičević T., Redovniković R.I. (2015). Evaluation of Toxicity and Biodegradability of Choline Chloride Based Deep Eutectic Solvents. Ecotoxicol. Environ. Saf..

[4] Mena I.F., Diaz E., Palomar J., Rodriguez J.J., Mohedano A.F. (2020). Cation and Anion Effect on the Biodegradability and Toxicity of Imidazolium– and Choline–Based Ionic Liquids. Chemosphere.

[5] Hayyan M. (2025). The Environmental Origin and Fate of Deep Eutectic Solvents: A Two-Edged Sword in Sustainability Claims. J. Mol. Liq..

[6] Zaib Q., Eckelman M.J., Yang Y., Kyung D. (2022). Are Deep Eutectic Solvents Really Green?: A Life-Cycle Perspective. Green Chem..

[7] Lobo Ferreira A.I.M.C., Vilas-Boas S.M., Silva R.M.A., Martins M.A.R., Abranches D.O., Soares-Santos P.C.R., Almeida Paz F.A., Ferreira O., Pinho S.P., Santos L.M.N.B.F. (2022). Extensive Characterization of Choline Chloride and Its Solid–Liquid Equilibrium with Water. Phys. Chem. Chem. Phys..

[8] Nejrotti S., Antenucci A., Pontremoli C., Gontrani L., Barbero N., Carbone M., Bonomo M. (2022). Critical Assessment of the Sustainability of Deep Eutectic Solvents: A Case Study on Six Choline Chloride-Based Mixtures. ACS Omega.

[9] Abbott A.P., Capper G., Davies D.L., Rasheed R.K., Tambyrajah V. (2003). Novel Solvent Properties of Choline Chloride/Urea Mixtures. Chem. Commun..

[10] Zhao H., Baker G.A., Holmes S. (2011). Protease Activation in Glycerol-Based Deep Eutectic Solvents. J. Mol. Catal. B Enzym..

[11] Mero A., Koutsoumpos S., Giannios P., Stavrakas I., Moutzouris K., Mezzetta A., Guazzelli L. (2023). Comparison of Physicochemical and Thermal Properties of Choline Chloride and Betaine-Based Deep Eutectic Solvents: The Influence of Hydrogen Bond Acceptor and Hydrogen Bond Donor Nature and Their Molar Ratios. J. Mol. Liq..

[12] Costa R., Brandão A.T.S.C., Pereira C.M., Silva A.F. (2023). Electrified Interfaces of Deep Eutectic Solvents. Electrochim. Acta.

[13] Lauw Y., Horne M.D., Rodopoulos T., Nelson A., Leermakers F.A.M. (2010). Electrical Double-Layer Capacitance in Room Temperature Ionic Liquids: Ion-Size and Specific Adsorption Effects. J. Phys. Chem. B.

[14] Jitvisate M. (2024). Direct Measurement of the Differential Capacitance of Deep Eutectic Solvents on Platinum and Glassy Carbon Electrodes. J. Phys. Chem. Lett..

[15] Ferreira E.S.C., Voroshylova I.V., Cordeiro M.N.D.S. (2024). Probing the Interface of Choline Chloride-Based Deep Eutectic Solvent Ethaline with Gold Surfaces: A Molecular Dynamics Simulation Study. Surf. Interfaces.

[16] Azmi S., Koudahi M.F., Frackowiak E. (2022). Reline Deep Eutectic Solvent as a Green Electrolyte for Electrochemical Energy Storage Applications. Energy Environ. Sci..

[17] Wang W., Sabugaa M.M., Chandra S., Asmara Y.P., Alreda B.A., Ulloa N., Elmasry Y., Kadhim M.M. (2023). Choline Chloride-Based Deep Eutectic Solvents as Electrolytes for Wide Temperature Range Supercapacitors. J. Energy Storage.

[18] Agieienko V., Buchner R. (2021). A Comprehensive Study of Density, Viscosity, and Electrical Conductivity of (Choline Chloride + Glycerol) Deep Eutectic Solvent and Its Mixtures with Dimethyl Sulfoxide. J. Chem. Eng. Data.

[19] Zhong M., Tang Q.F., Zhu Y.W., Chen X.Y., Zhang Z.J. (2020). An Alternative Electrolyte of Deep Eutectic Solvent by Choline Chloride and Ethylene Glycol for Wide Temperature Range Supercapacitors. J. Power Sources.

[20] Hayler H.J., Perkin S. (2022). The Eutectic Point in Choline Chloride and Ethylene Glycol Mixtures. Chem. Commun..

[21] Petrouleas V., Lemmon R.M. (1978). Calorimetric Studies of Choline Chloride, Bromide, and Iodide. J. Chem. Phys..

[22] Shinwari M.W., Zhitomirsky D., Deen I.A., Selvaganapathy P.R., Deen M.J., Landheer D. (2010). Microfabricated Reference Electrodes and Their Biosensing Applications. Sensors.

[23] Silva F., Gomes C., Figueiredo M., Costa R., Martins A., Pereira C.M. (2008). The Electrical Double Layer at the [BMIM][PF6] Ionic Liquid/Electrode Interface—Effect of Temperature on the Differential Capacitance. J. Electroanal. Chem..

[24] Gateman S.M., Gharbi O., Gomes de Melo H., Ngo K., Turmine M., Vivier V. (2022). On the Use of a Constant Phase Element (CPE) in Electrochemistry. Curr. Opin. Electrochem..

[25] Pajkossy T., Müller C., Jacob T. (2018). The Metal–Ionic Liquid Interface as Characterized by Impedance Spectroscopy and in Situ Scanning Tunneling Microscopy. Phys. Chem. Chem. Phys..

[26] Reuter D., Münzner P., Gainaru C., Lunkenheimer P., Loidl A., Böhmer R. (2021). Translational and Reorientational Dynamics in Deep Eutectic Solvents. J. Chem. Phys..

[27] Lapeña D., Bergua F., Lomba L., Giner B., Lafuente C. (2020). A Comprehensive Study of the Thermophysical Properties of Reline and Hydrated Reline. J. Mol. Liq..

[28] Wu J., Liu S., Tan Z., Guo Y., Zhou J., Mao B., Yan J. (2021). Effect of Hydrogen Bond Donor Molecules Ethylene Glycerol and Lactic Acid on Electrochemical Interfaces in Choline Chloride Based-Deep Eutectic Solvents. J. Chem. Phys..

[29] Mamme M.H., Moors S.L.C., Terryn H., Deconinck J., Ustarroz J., De Proft F. (2018). Atomistic Insight into the Electrochemical Double Layer of Choline Chloride–Urea Deep Eutectic Solvents: Clustered Interfacial Structuring. J. Phys. Chem. Lett..

[30] Hammond O.S., Li H., Westermann C., Al-Murshedi A.Y.M., Endres F., Abbott A.P., Warr G.G., Edler K.J., Atkin R. (2019). Nanostructure of the Deep Eutectic Solvent/Platinum Electrode Interface as a Function of Potential and Water Content. Nanoscale Horiz..

[31] Kaur S., Sharma S., Kashyap H.K. (2017). Bulk and Interfacial Structures of Reline Deep Eutectic Solvent: A Molecular Dynamics Study. J. Chem. Phys..

[32] Chen Z., McLean B., Ludwig M., Stefanovic R., Warr G.G., Webber G.B., Page A.J., Atkin R. (2016). Nanostructure of Deep Eutectic Solvents at Graphite Electrode Interfaces as a Function of Potential. J. Phys. Chem. C.

[33] Brug G.J., van den Eeden A.L.G., Sluyters-Rehbach M., Sluyters J.H. (1984). The Analysis of Electrode Impedances Complicated by the Presence of a Constant Phase Element. J. Electroanal. Chem. Interfacial Electrochem..

[34] Dean W., Klein J., Gurkan B. (2021). Do Deep Eutectic Solvents Behave Like Ionic Liquid Electrolytes? A Perspective from the Electrode-Electrolyte Interface. J. Electrochem. Soc..

[35] Harifi-Mood A.R., Buchner R. (2017). Density, Viscosity, and Conductivity of Choline Chloride + Ethylene Glycol as a Deep Eutectic Solvent and Its Binary Mixtures with Dimethyl Sulfoxide. J. Mol. Liq..

[36] Abbott A.P., Harris R.C., Ryder K.S. (2007). Application of Hole Theory to Define Ionic Liquids by Their Transport Properties. J. Phys. Chem. B.

[37] Wu J., Liang Q., Yu X., Lü Q., Ma L., Qin X., Chen G., Li B. (2021). Deep Eutectic Solvents for Boosting Electrochemical Energy Storage and Conversion: A Review and Perspective. Adv. Funct. Mater..

[38] Agieienko V., Buchner R. (2019). Densities, Viscosities, and Electrical Conductivities of Pure Anhydrous Reline and Its Mixtures with Water in the Temperature Range (293.15 to 338.15) K. J. Chem. Eng. Data.

[39] Costa R., Figueiredo M., Pereira C.M., Silva F. (2010). Electrochemical Double Layer at the Interfaces of Hg/Choline Chloride Based Solvents. Electrochim. Acta.

[40] Figueiredo M., Gomes C., Costa R., Martins A., Pereira C.M., Silva F. (2009). Differential Capacity of a Deep Eutectic Solvent Based on Choline Chloride and Glycerol on Solid Electrodes. Electrochim. Acta.

[41] Yuan C., Zhang X., Ren Y., Feng S., Liu J., Wang J., Su L. (2019). Temperature- and Pressure-Induced Phase Transitions of Choline Chloride-Urea Deep Eutectic Solvent. J. Mol. Liq..

[42] Waskom M. (2021). Seaborn: Statistical Data Visualization. J. Open Source Softw..

[43] Awasthi H., Renuka H., Srivastava A.K., Goel S. (2023). Laser-Induced Graphene-Based Flexible Interdigital Electrode Realizing Micro Supercapacitor Sustainable in Different Temperatures. Energy Storage.

[44] Nolasco M.M., Pedro S.N., Vilela C., Vaz P.D., Ribeiro-Claro P., Rudić S., Parker S.F., Freire C.S.R., Freire M.G., Silvestre A.J.D. (2022). Water in Deep Eutectic Solvents: New Insights From Inelastic Neutron Scattering Spectroscopy. Front. Phys..

[45] Murbach M., Gerwe B., Dawson-Elli N., Tsui L. (2020). Impedance.Py: A Python Package for Electrochemical Impedance Analysis. J. Open Source Softw..

[46] Lebigot E.O. Uncertainties: A Python Package for Calculations with Uncertainties. https://pypi.org/project/uncertainties/.

